# Validity and Reliability of Brief International Cognitive Assessment for Multiple Sclerosis (BICAMS) in Indonesia and the Correlation with Quality of Life

**DOI:** 10.1155/2019/4290352

**Published:** 2019-05-23

**Authors:** Riwanti Estiasari, Yuhyi Fajrina, Diatri Nari Lastri, Syarli Melani, Kartika Maharani, Darma Imran, David Pangeran, Freddy Sitorus

**Affiliations:** ^1^Department of Neurology, Faculty of Medicine Universitas Indonesia, Indonesia; ^2^Dr. Cipto Mangunkusumo General Hospital, Jakarta, Indonesia

## Abstract

**Introduction:**

Multiple Sclerosis (MS) can affect cognitive function that might interfere with quality of life. Processing speed and memory are the most common area of cognitive impairment. Cognitive evaluation in daily practice is often difficult to be performed since it needs neuropsychological expert and is time-consuming. Brief International Cognitive Assessment for MS (BICAMS) is valid and practical for cognitive evaluation. This study aims to validate BICAMS in Indonesian MS patients and healthy controls (HC) and to analyse the effect of cognitive impairment on quality of life.

**Methods:**

BICAMS, which composes Symbol Digits Modalities Test (SDMT), California Verbal Learning Test-Second Edition (CVLT-II), and Brief Visuospatial Memory Test-Revised (BVMT-R), was translated and cross-culturally adapted to Indonesian from the original BICAMS and then administered to 40 Indonesian MS patients and 66 HC matched by sex, age, and education. Test-retest reliability was performed on 16-MS patients and 42 HC. Quality of life was measured using Multiple Sclerosis Quality of Life (MSQOL-54) instrument.

**Results:**

The SDMT, CVLT-II, and BVMT-R score in MS patients were significantly lower than those in HC (effect size, r: 0.61, 0.36, and 0.47, respectively). Test-retest reliability for all tests was satisfactory with correlation coefficient for SDMT, CVLT-II, and BVMT-R in MS subjects 0.86, 0.81, and 0.83, respectively. Using 5th percentile of HC score as cut-off, 15% MS subjects had impairment in one test, 27.5% in two tests, and 40% in three tests. BICAMS was moderately correlated with EDSS but was not correlated with disease duration and relapse rate. SDMT score correlated with physical function and physical and mental role limitation.

**Conclusion:**

BICAMS is valid and reliable for assessing cognitive function of Indonesia MS patients.

## 1. Introduction

Multiple sclerosis (MS) is an autoimmune inflammatory central nervous system disorder characterized by demyelinating lesion. MS mostly affects young adult which typically begins at 20-40 years old. It is more prevalent in female compared to man. In addition, it is the most common disabling disorder in young people [1].

MS does not only affect physical function but also cognitive function, which may impact on reduced productivity and quality of life (QOL) [2]. Cognitive impairment can affect physical independence and medication adherence [3]. It is detected in 41% MS patients, of which 28% experience significant decline in 5 years [4]. Deficit in processing speed [5] and memory [6, 7] are the most common cognitive impairment in MS and more prevalent in progressive type than relapsing-remitting MS (RRMS) [8]. However, the deficit can also be detected in the early onset of MS (clinically isolated syndrome, CIS) [9].

One challenge regarding the assessment of cognitive function is that neuropsychology assessment usually needs special expertise and is time-consuming, which hinders its application in daily practice. This situation hampers the detection of cognitive impairment, especially the subtle ones, in MS.

In 2012, an expert panel proposed Brief International Cognitive Assessment for Multiple Sclerosis (BICAMS) which focuses on cognitive processing speed and verbal and visual memory. BICAMS consists of three tests: Symbol Digits Modalities Test (SDMT) assessing processing speed/working memory, California Verbal Learning Test-Second Edition (CVLT-II) assessing learning and verbal memory, and Brief Visuospatial Memory Test-Revised (BVMT-R) assessing learning and visuospatial memory [10]. It has been validated in several languages including non-English speaking countries. These studies showed that BICAMS is valid and reliable and can be easily performed. It can be carried out by nontrained healthcare staff and has been applied in many countries [11–19].

The objectives of our study were to validate and assess the reliability of BICAMS in Indonesia language and to evaluate the association between cognitive impairment with disease duration, relapse frequency, and physical disability. The secondary objective of this study was to observe the impact of cognitive impairment on quality of life using the MSQOL-54 instrument.

## 2. Materials and Methods

### 2.1. Study Design and Participants

A cross-sectional study was performed in Dr. Cipto Mangunkusumo General Hospital Jakarta (RSCM) from September 2017 to June 2018. This study was approved by the Ethics Committee Faculty of Medicine Universitas Indonesia (Number:0381/UN2. F1/ETIK/2018).

We invited MS patients who had been registered in RSCM to participate in this study. Forty MS patients diagnosed using McDonald 2010 criteria [20] and 66 healthy control (HC) were recruited in this study consecutively. The inclusion criteria were age over 18 years and being able to read and write in Indonesia and written consent for all procedures was provided. Exclusion criteria applied were having other neurological disorders including stroke, epilepsy, brain tumour, brain infection, and history of brain injury; history of major depression; receiving substance influencing psychiatric status including alcohol and having severe visual and motor disability that can interfere in the cognitive test. HC were recruited from population around our clinics, relatives, or friends of MS patients that were not affected with MS and fulfilled the same criteria. HC were matched with MS subjects based on age, sex, and education level.

### 2.2. Procedure

We followed the BICAMS international standard validation procedure that consists of 5 steps: (1) standardization and translation of test stimuli; (2) standardization and translation of test instructions; (3) normalization; (4) test-retest reliability; (5) criterion-related validity [21].

BICAMS was translated into Indonesian by two different professional native speaking Indonesian translators blinded to each other. Words for CVLT-II were adapted to Indonesia culture. Translated version was compared and different words were reconciliated among neurologists. A preliminary version was tested to 10 MS patients and 10 HC. We noted all difficulties during the trial session. The preliminary version was then back-translated into English by two different English native speakers blinded to each other. The final version was developed by selecting words that have the closest meaning to the original version and are familiar in large populations in Indonesia.

The tests were administered in fixed order: SDMT, CVLT-II, and BVMT-R. SDMT was used to assess processing speed. Subjects were presented with a sequence of 9 symbols. Each symbol was paired with a single digit displayed on the top of the page. Participants were given a short practice session to recognize the symbol and the digit pair. Thereafter, they were asked to decrypt the symbols to the associated digits as rapidly as possible in 90 seconds. The maximum score for this test was 110. We performed the oral version of SDMT [22].

CVLT-II was used to assess auditory/verbal learning. Initially, examiner read aloud a list of 16 words. Participants listened to the list and repeated it as many as they can memorize. The number of words recalled was recorded and the trial was repeated for 5 times. The highest score for this test was 80 [23].

BVMT-R was used to evaluate visual/spatial memory. Six abstract figures were shown to the participants for 10 seconds. The figures were then removed, and participants were asked to draw those figures on paper manual responses. The time for drawing is not limited. Each figure was scored 0, 1, and 2 based on accuracy and location. Three trials were performed, and the total score was calculated from the sum of all 3 trial scores. The maximum score for this test was 26 [24].

On the same day with BICAMS examination, subjects were asked to fill the MSQOL-54 questionnaire which has already been validated into Indonesian. MSQOL-54 was transculturally adapted by performing a standardized forward-backward method and continued with assessing the reliability, internal validation, and external validation (unpublished). From 40 MS subjects, 3 subjects refused to fill the MSQOL questionnaire. This questionnaire consists of 54 questions measuring physical health, emotional well-being, role limitations due to physical problems, role limitations due to emotional problems, pain, energy, health perceptions, social function, cognitive function, health distress, sexual function, satisfaction with sexual function, change in health, and overall quality of life [25].

Before the BICAMS examination and filling the MSQOL-54 questionnaire, demographic and clinical data were collected including education level, duration of illness, relapse frequency, and treatment. Neurological examination, EDSS, and depression evaluation using mini-ICD-10 questionnaire were also performed.

### 2.3. Statistical Analysis

Data distribution was analysed using Kolmogorov-Smirnov test. The difference between SDMT, CVLT-II, and BVMTR score in MS and HC subjects was examined using paired student T-test. Test-retest reliability was performed following 1-3 weeks and analysed using Pearson correlation coefficient. The r-values for test-retest correlation were considered adequate if >0.70 and good if >0.80. Criterion-related validity of BICAMS was assessed by analysing the correlation between age, EDSS, disease duration, and relapse rate to BICAMS score using Spearman correlation. Pearson correlation was used to analyse the correlation between quality of life and cognitive function. The p-value less than 0.05 was considered significant. Statistical analysis was performed using SPSS-25.0 and GraphPad Prism 5.

## 3. Results

There was no significant difference in sex, age, and education duration between MS and HC groups (Table 1). Among MS subjects, 31 (77.5%) were RRMS and 9 (22.5%) were SPMS (Secondary Progressive MS). The mean EDSS score was 3.3±2.1, in which higher score was observed in male compared to female (median (range)= 6 (2.5-7) vs. 2.5 (1-7.5), p=0.03). Depression was found in 13 MS subjects (32.5%) and none in HC group. Not all subjects can afford disease modifying drug (DMD) since it is very expensive in Indonesia. Only 10 (25%) patients used DMD (4 patients used Fingolimod and 6 patients used Interferon Beta IA). The most common treatment was azathioprine (42.5%). Eleven subjects (27.5%) decided not to take any medication.

In all tests, MS subjects had significantly lower scores than HC (Table 2). The effect size was highest in SDMT (effect size r=0.61) followed by BVMT-R (0.47) and CVLT-II (0.36). The Cronbach-*α* coefficients in MS and HC group for all tests were 0.78 and 0.61, respectively.

In MS group, male subjects had lower score compared to female with significant differences in SDMT and CVLT-II. In HC group, the difference was not apparent. In reverse, the effect of education was only observed in SDMT score on HC group (p=0.05). Negative correlation was also found between age and SDMT (r=-0.27; p=0.03) and BVMT-R score (r=-0.28; p=0.02) only on HC (Table 3).

All BICAMS scores were significantly lower in SPMS subtype subjects. All tests also showed significant negative correlation with EDSS, but this was not found on the relation to disease duration and relapse rate (Table 3).

Test-retest reliability was performed on 16 MS patients and 42 HC (Table 4). Both groups showed significant correlation between test and retest, but the correlation in HC was weaker than those in MS.

Using the 5^th^ percentile of HC score tests as cut off point, the test score of MS subjects was classified into impaired and not impaired. SDMT, CVLT-II, and BVMT-R impairment were found on 20 (50.0%), 13 (27.5%), and 11 (32.5%) subjects, respectively. Impairment in 1, 2, and 3 tests was shown in 6 (15.0%), 11 (27.5%), and 16 (40%) subjects, respectively.

The impact of cognitive impairment on QOL was mostly seen in physical functions. SDMT, CVLT-II, and BVMT-R score significantly correlated with physical function (r=0.43, 0.43, and 0.45, respectively). SDMT also correlated with role limitation due to both physical and emotional problems (Table 5).

## 4. Discussion

Indonesia is a tropical country in which the prevalence of MS is estimated low [26]. Previously MS diagnosis often cannot be established due to facility limitation, mainly* magnetic resonance imaging *(MRI). Nowadays, MRI is becoming more and more available in many cities in Indonesia. Knowledge and concern for MS are also increasing among neurologist and other medical practitioners. As a result, the number of MS cases also increases even though it is still considered rare in our country. Comprehensive treatment for MS in Indonesia remains far from satisfaction and cognitive evaluation is often overlooked. With a high burden of clinical work, practical and short cognitive battery that can be administered by a physician is needed since not all neurology clinics in Indonesia have Neuropsychology expert.

There are several batteries proposed for assessing cognitive function in MS. Minimal Assessment of Cognitive Function in MS (MACFIMS) contains seven neuropsychology tools that cover five domains of cognitive function [27]. Another tool is Brief Repeatable Battery-Neuropsychology (BRB-N) test, which has also been widely used in clinical practice. Selective Reminding Test (SRT), the 10/36 Spatial Recall Test (10/36), the Symbol Digit Modalities Test (SDMT), the Paced Auditory Serial Addition Task 2 and 3 seconds (PASAT 2-3), and the Word List Generation (WLG) were included in BRB-N [28]. Both MACFIMS and BRB-N need more than one hour to be administered.

BICAMS is a brief cognitive evaluation tool that is recommended for MS and can be used worldwide. It is valid and reliable with high sensitivity and specificity. BICAMS can be administered by a physician for only 15 minutes [10]. In this study, BICAMS was translated and culturally adapted into Indonesian.

This study found significantly lower score in all BICAMS tests on MS than HC (p<0.0001). SDMT has the largest effect size (r=0.61), followed by BVMT-R (r=0.47). This result was similar with previous studies which reported that SDMT and BVMT-R were more sensitive in distinguishing the cognition in MS and healthy subjects [13, 14, 29]. Different result was reported in Brazilian study which found that the effect size of BVMT-R was lower compared with SDMT and CVLT-II [11].

Our study found good correlation between test and retest score in SDMT (r=0.86), CVLT-II (r=0.81), and BVMT-R (r=0.83). In the HC group, the strongest correlation was observed in SDMT (r=0.76). BVMT-R and CVLT-II had weaker correlation (r=0.51 and 0.49). This result showed that BICAMS is reliable for assessing cognitive function in Indonesian MS patients.

Based on MS subtype, SPMS patients in this study had significantly lower score in all tests compared with RRMS. This result was in line with the negative correlation between EDSS and BICAMS score in those subjects. This proved the criterion-related validity of BICAMS for Indonesian MS patients. Studies in Japan and Brazil also found correlation between EDSS, disease duration, and BICAMS score [11, 14]. However, our study did not find correlation between BICAMS score and disease duration, which was similar with the result from Lithuania [12].

Based on our result, SDMT and CVLT-II were affected by sex in the MS group, but it was not found in HC. This result was in conjunction with the correlation to the EDSS score since MS male subjects had higher EDSS score compared to female. This study did not find association between age, education, and BICAMS score in MS subjects, which was partially seen in HC groups. This might be due to the distribution of age and education in our subjects, most of which were younger than 40 year and had high education.

The prevalence of cognitive impairment on each test using 5^th^ percentile of HC score in Indonesian patients was similar to the Turkish and Greek population [13, 15]. However, if the impairment was classified based on the number of impaired tests, our subjects had higher impairment in three tests compared with Turkish and Greek ones. This difference was speculated due to no CIS subjects that were included in our study.

In this study, we found significant correlation between SDMT score with physical functioning and role limitation due to physical and mental problems. CVLT-II and BVMTR only significantly correlated with physical functioning. This finding was different with Sandi et al. who found significant correlation between BICAMS score with cognitive, physical, and social functioning subscales, overall quality of life and general health subscales, sexual function, and satisfaction with sexual function [16]. Another study by Hoog et al. analyzed the correlation between QOL using Sick Impact Profile (SIP) and cognitive function using MACFIMS. Logistics regression model showed that EDSS and SDMT performance could predict the poor physical health-related quality of life (HRQOL). SDMT alone could also predict poor overall HRQOL and psychosocial HRQOL. Even though our result failed in showing the correlation between cognitive function and both social aspect and overall quality of life, it supported the evidence that SDMT score had an association with the physical aspect of QOL [30]. Cultural background might also influence the result of QOL since the MSQOL-54 is a self-filling instrument.

There were numbers of limitation in our study including a small sample size. Patients with relapse during the study were not excluded, and we have two patients who received high dose of methylprednisolone recently. We used the same form for CVLT-II retest which may lead to the practice effect. An alternate form is needed for the implementation of BICAMS in Indonesia. In this study we did not exclude subjects with depression and did not screened for fatigue status. However, there was no significant BICAMS score difference between MS subjects with and without depression. Fatigue might influence the subjects' performance during the test as another study showed significant correlation with SDMT score [17].

## 5. Conclusions

BICAMS is valid and reliable for Indonesia MS patients which is short and easy to be performed. It can be widely used in Indonesia. Correlation between cognitive function and QOL described the importance of cognitive evaluation in MS care.

## Figures and Tables

**Table 1:** Demographic and clinical characteristics of MS and healthy control subjects.

	MS (n=40)	Healthy control (n=66)	*p*
Sex – N(%)			0.25^#^
Female	33 (82.5)	48 (72.7)	
Male	7 (17.5)	18 (27.3)	
Education -N (%)			0.05*∗*
≤12 years	10 (25.0)	7 (10.6)	
>12 years	30 (75.0)	59 (89.4)	
Age (years) – Median (range)	31 (20-61)	29 (22-51)	0.15^∧^
EDSS – Median (range)	3 (1-7.5)		
Depression -N(%)	13 (32.5)		
Duration of illness (years)	4 (0.1-15)		

#Fisher exact test, *∗*Chi-Square test, ^∧^Wilcoxon sign ranked test

**Table 2:** Comparison of BICAMS score between MS and HC subjects.

	MS	HC	*p∗*
	Mean±SD		
SDMT	40.9±14.8	64.8±16.2	<0.0001
SDMT retest	42.7±15.2	74.3±15.5	<0.0001
Effect size (r)	0.61		

CVLT-II	52.0±12.8	61.5±9.7	<0.0001
CVLT-II retest	61.5±14.3	73.5±61.5	0.005
Effect size (r)	0.36		

BVMT-R	22.2±7.7	29.3±5.6	<0.0001
BVMT-R retest	25.8±8.6	33.4±2.8	0.003
Effect size (r)	0.47		

*∗*Paired T-test

**Table 3:** Association of BICAMS scores with sex, education, depression, age, MS subtype, EDSS, disease duration, and relapse rate.

	Multiple Sclerosis (N=40)			Healthy Control (N=66)		
	SDMT	CVLT-II	BVMT-R	SDMT	CVLT-II	BVMT-R
Sex - median (range)						
Female	48 (12-69)	56 (25-71)	25 (4-34)	62 (40-110)	64.5(36-77)	31.5(14-36)
Male	30 (8-49)	34 (23-72)	18 (7-32)	64.5(42-92)	60(43-75)	30 (17-36)
*p∗*	*0.05*	*0.03*	0.15	0.92	0.41	0.58

Education - median (range)						
>12 years	47 (15-69)	54 (23-72)	24.5(4-32)	63 (42-110)	63 (36-77)	31 (14-36)
≤12 years	34 (8-57)	54.5 (25-64)	20.5 (6-34)	53 (40-71)	62 (37-74)	28 (19-33)
*p∗*	0.10	0.47	0.36	*0.05*	0.69	0.14

Depression–median (range)						
Yes	36 (12-57)	56 (25-64)	23 (4-34)			
No	46 (8-69)	46 (8-69)	24 (7-32)			
*p∗*	0.98	0.83	0.89			

Age^∧^ - r (p)	-0.004(0.98)	-0.11 (0.5)	0.02(0.89)	-0.27(*0.03*)	-0.11(0.39)	-0.28*(0.02*)

MS Subtype – median (range)						
RRMS (N=31)	49 (8-69)	58 (23-72)	26 (4-34)			
SPMS (N=9)	29 (12-39)	42 (25-68)	17 (6-29)			
*p∗*	*<0.001*	*0.018*	*0.015*			

EDSS^∧^*r(p)*	-0.5*(<0.001*)	-0.46(*0.002*)	-0.49(*0.001*)			

Disease duration^∧^*r(p)*	-0.23 (0.15)	-0.19 (0.23)	-0.18 (0.27)			

Relapse rate^∧^*r(p)*	0.04 (0.78)	-0.15 (0.93)	0.18 (0.25)			

*∗*Mann Whitney U Test; ^∧^Spearman Correlation

**Table 4:** Test-retest reliability.

	SDMT		CVLT-II		BVMT-R	
	r	p	r	p	r	p
All subjects	0.88	<0.0001	0.75	<0.0001	0.77	<0.0001
MS Patient	0.86	<0.0001	0.81	<0.0001	0.83	<0.0001
Healthy control	0.76	<0.0001	0.49	0.01	0.51	0.008

**Table 5:** Correlation between MSQOL-54 item score and BICAMS score.

	SDMT		CVLT-II		BVMT-R	
	r*∗*	p	r*∗*	p	r*∗*	p
Physical function	0.43	*0.009*	0.43	*0.008*	0.45	*0.006*
Health perceptions	-0.14	0.4	-0.18	0.27	-0.20	0.24
Energy/fatigue	-0.14	0.4	-0.21	0.22	-0.18	0.27
Role limitations - physical	0.34	*0.04*	0.21	0.21	0.20	0.23
Pain	-0.03	0.8	-0.19	0.25	-0.12	0.47
Sexual function	-0.12	0.7	0.30	0.92	-0.43	0.14
Social function	-0.3	0.2	-0.15	0.36	-0.23	0.17
Health distress	-0.12	0.5	-0.10	0.56	-0.18	0.27
Overall quality of life	-0.14	0.41	-0.12	0.48	-0.14	0.41
Emotional well-being	-0.07	0.7	-0.07	0.67	-0.20	0.24
Role limitation-emotional	0.36	*0.03*	-0.001	0.99	0.14	0.42
Cognitive function	0.19	0.25	-0.38	0.83	0.10	0.55
PHC	0.12	0.47	0.07	0.68	0.02	0.88
MHC	0.07	0.67	0.005	0.97	-0.07	0.68

*∗*Spearman Correlation; PHC: Physical Health Composite; MHC: Mental Health Composite

## Data Availability

The data used to support the findings of this study are available from the corresponding author upon request.

## References

[1] Thompson A. J., Baranzini S. E., Geurts J., Hemmer B., Ciccarelli O. (2018). Multiple sclerosis. *The Lancet*.

[2] Islas MAM., Ciampi E. (2019). Assessment and impact of cognitive impairment in multiple sclerosis: an overview. *Biomedicines*.

[3] Langdon D. W. (2011). Cognition in multiple sclerosis. *Current Opinion in Neurology*.

[4] Eijlers A. J. C., van Geest Q., Dekker I. (2018). Predicting cognitive decline in multiple sclerosis: a 5-year follow-up study. *Brain*.

[5] Manca R., Sharrack B., Paling D., Wilkinson ID., Venneri A. (2018). Brain connectivity and cognitive processing speed in multiple sclerosis: a systematic review. *J Neurol Sci*.

[6] Deluca J., Leavitt V. M., Chiaravalloti N., Wylie G. (2013). Memory impairment in multiple sclerosis is due to a core deficit in initial learning. *Journal of Neurology*.

[7] Chiaravalloti N. D., DeLuca J. (2008). Cognitive impairment in multiple sclerosis. *The Lancet Neurology*.

[8] Sumowski J. F., Benedict R., Enzinger C. (2018). Cognition in multiple sclerosis: State of the field and priorities for the future. *Neurology*.

[9] Baysal Kıraç L., Ekmekçi Ö., Yüceyar N., Sağduyu Kocaman A. (2014). Assessment of early cognitive impairment in patients with clinically isolated syndromes and multiple sclerosis. *Behavioural Neurology*.

[10] Langdon D. W., Amato M. P., Boringa J. (2012). Recommendations for a brief international cognitive assessment for multiple sclerosis (BICAMS). *Multiple Sclerosis Journal*.

[11] Spedo C. T., Frndak S. E., Marques V. D. (2015). Cross-cultural adaptation, reliability, and validity of the BICAMS in Brazil. *The Clinical Neuropsychologist*.

[12] Giedraitienė N., Kizlaitienė R., Kaubrys G. (2015). The BICAMS battery for assessment of Lithuanian-speaking multiple sclerosis patients: Relationship with age, education, disease disability, and duration. *Medical Science Monitor*.

[13] Polychroniadou E., Bakirtzis C., Langdon D. (2016). Validation of the Brief International Cognitive Assessment for Multiple Sclerosis (BICAMS) in Greek population with multiple sclerosis. *Multiple Sclerosis and Related Disorders*.

[14] Niino M., Fukazawa T., Kira J. (2017). Validation of the brief international cognitive assessment for multiple sclerosis in japan. *Multiple Sclerosis Journal – Experimental, Translational and Clinical*.

[15] Ozakbas S., Yigit P., Cinar B. P., Limoncu H., Kahraman T., Kösehasanogullari G. (2017). The Turkish validation of the Brief International Cognitive Assessment for Multiple Sclerosis (BICAMS) battery. *BMC Neurology*.

[16] Sandi D., Rudisch T., Füvesi J. (2015). The Hungarian validation of the brief international cognitive assessment for multiple sclerosis (BICAMS) battery and the correlation of cognitive impairment with fatigue and quality of life. *Multiple Sclerosis and Related Disorders*.

[17] Sousa C., Rigueiro-Neves M., Miranda T. (2018). Validation of the brief international cognitive assessment for multiple sclerosis (BICAMS) in the Portuguese population with multiple sclerosis. *BMC Neurology*.

[18] O'Connell K., Langdon D., Tubridy N., Hutchinson M., McGuigan C. (2015). A preliminary validation of the brief international cognitive assessment for multiple sclerosis (BICAMS) tool in an Irish population with multiple sclerosis (MS). *Multiple Sclerosis and Related Disorders*.

[19] Dusankova J. B., Kalincik T., Havrdova E., Benedict R. H. B. (2012). Cross cultural validation of the minimal assessment of cognitive function in multiple sclerosis (MACFIMS) and the brief international cognitive assessment for multiple sclerosis (BICAMS). *The Clinical Neuropsychologist*.

[20] Polman C. H., Reingold S. C., Banwell B. (2011). Diagnostic criteria for multiple sclerosis: 2010 revisions to the McDonald criteria. *Annals of Neurology*.

[21] Benedict R. H., Amato M. P., Boringa J. (2012). Brief International Cognitive Assessment for MS (BICAMS): international standards for validation. *BMC Neurology*.

[22] Smith A. (1982). *Symbol digit modalities test: Manual*.

[23] Delis D. C., Kramer J. H., Kaplan E. (2000). *California Verbal Learning Test Manual: Second Edition, Adult Version*.

[24] Benedict R. H. (1997). *Brief Visuospatial Memory Test-Revised: Professional Manual*.

[25] Vickrey B. G., Hays R. D., Harooni R., Myers L. W., Ellison G. W. (1995). A health-related quality of life measure for multiple sclerosis. *Quality of Life Research*.

[26] Atlas-of-MS.pdf [Internet]. [cited 2018 Nov 20]. https://www.msif.org/wp-content/uploads/2014/09/Atlas-of-MS.pdf.

[27] Benedict R. H. B., Fischer J. S., Archibald C. J. (2002). Minimal neuropsychological assessment of ms patients: a consensus approach. *Clin Neuropsychol*.

[28] Sepulcre J., Vannotti S., Hernández R. (2006). Cognitive impairment in patients with multiple sclerosis using the Brief Repeatable Battery-Neuropsychology test. *Multiple Sclerosis Journal*.

[29] Walker L. A. S., Osman L., Berard J. A., Rees L. M., Freedman M. S., MacLean H. (2016). Brief International Cognitive Assessment for Multiple Sclerosis (BICAMS): Canadian contribution to the international validation project. *Journal of the Neurological Sciences*.

[30] Hoogs M., Kaur S., Smerbeck A. (2011). Cognition and physical disability in predicting health-related quality of life in multiple sclerosis. *International Jounal of MS Care*.

